# A Web Resource for Standardized Benchmark Datasets, Metrics, and Rosetta Protocols for Macromolecular Modeling and Design

**DOI:** 10.1371/journal.pone.0130433

**Published:** 2015-09-03

**Authors:** Shane Ó Conchúir, Kyle A. Barlow, Roland A. Pache, Noah Ollikainen, Kale Kundert, Matthew J. O'Meara, Colin A. Smith, Tanja Kortemme

**Affiliations:** 1 California Institute for Quantitative Biosciences (QB3), University of California San Francisco, San Francisco, California, United States of America; 2 Department of Bioengineering and Therapeutic Sciences, University of California San Francisco, San Francisco, California, United States of America; 3 Graduate Program in Bioinformatics, University of California San Francisco, San Francisco, California, United States of America; 4 Graduate Program in Biophysics, University of California San Francisco, San Francisco, California, United States of America; 5 Department of Pharmaceutical Chemistry, University of California San Francisco, San Francisco, California, United States of America; University of Michigan, UNITED STATES

## Abstract

The development and validation of computational macromolecular modeling and design methods depend on suitable benchmark datasets and informative metrics for comparing protocols. In addition, if a method is intended to be adopted broadly in diverse biological applications, there needs to be information on appropriate parameters for each protocol, as well as metrics describing the expected accuracy compared to experimental data. In certain disciplines, there exist established benchmarks and public resources where experts in a particular methodology are encouraged to supply their most efficient implementation of each particular benchmark. We aim to provide such a resource for protocols in macromolecular modeling and design. We present a freely accessible web resource (https://kortemmelab.ucsf.edu/benchmarks) to guide the development of protocols for protein modeling and design. The site provides benchmark datasets and metrics to compare the performance of a variety of modeling protocols using different computational sampling methods and energy functions, providing a “best practice” set of parameters for each method. Each benchmark has an associated downloadable benchmark capture archive containing the input files, analysis scripts, and tutorials for running the benchmark. The captures may be run with any suitable modeling method; we supply command lines for running the benchmarks using the Rosetta software suite. We have compiled initial benchmarks for the resource spanning three key areas: prediction of energetic effects of mutations, protein design, and protein structure prediction, each with associated state-of-the-art modeling protocols. With the help of the wider macromolecular modeling community, we hope to expand the variety of benchmarks included on the website and continue to evaluate new iterations of current methods as they become available.

## Introduction

Structure-based modeling and design of biological macromolecules have become rich areas of computational research and method development [1–5]. The accuracy of these modeling protocols on diverse applications can be assessed via use of increasingly available, high quality curated experimental datasets [6–10]. Demonstration of the utility of a new prediction or design method requires, at the very least, a proof-of-concept case that exhibits initial success. Further widespread adoption of the method requires more extensive validation: demonstrated success and careful evaluation of key limitations on multiple, diverse, test cases. This general utility can be shown through the use of a suitable benchmark set.

Even though the compilation of these benchmarks is often essential to the creation of novel computational methods, the successful application of a method can often overshadow the critical role of benchmarking during its development. Furthermore, the associated publication of a new method may not contain a description of the dataset or statistical analysis in a format that is readily usable for developers of alternate methods, creating additional obstacles for a direct comparison. Organizations such as CASP [11] and CAPRI [12] create blind prediction tests for problems in protein structure prediction, protein-protein docking, and other applications, but many questions in the field of macromolecular modeling and design could also benefit from canonical benchmarks such as those that exist for protein-protein docking [10,13]. To facilitate rapid, iterative development, it is convenient to make benchmarks available for retrospective testing (although it is essential to pay attention to issues of overfitting to a particular target problem, even for large and diverse datasets).

Even in cases where an effective benchmark has been defined and the efficacy of a modeling protocol has been measured and published, it may be difficult to reproduce similar results post-publication as the method evolves. Protocols in large, complex software suites, such as Rosetta, are highly dependent on core functionality. For example, a sampling algorithm may yield varying results as changes are made to its accompanying score function. Regular benchmarking to track changes in performance is desirable both when core functionality is altered and when the specific protocol has been modified directly. To determine what constitutes the best practice, a user needs access to current benchmarking results, or at the very least, clear instructions on how to benchmark against the latest version of the protocol.

Here we present a web resource (https://kortemmelab.ucsf.edu/benchmarks) to address some of the aforementioned difficulties associated with informative benchmarking. We define the following criteria for a benchmark set in this resource: First, the scientific question or modeling problem posed by the benchmark must be clearly defined. Second, the input dataset should contain numerous, varied test cases that cover a broad range of possible inputs a user might use in a protocol. Success is easier to find when only a small subset of potential test cases is employed; a more general set indicates a correspondingly more generally useful method, and ameliorates issues with over-fitting a method to perform well on a specific test case. To be suitable for comparison against predictions, this input data set should be made up of experimentally validated data (we will refer to predicted data as “predictions” and experimentally determined data as “experiments”). Third, instructions on how to run each computational method should be provided with enough detail and clarity such that researchers other than the developers of a given method are able to use the resource. Finally, each benchmark set should be accompanied both by an appropriate set of defined metrics to quantify how successfully the method addresses the modeling problem and by a set of analysis tools which, given input in a defined format, computes these metrics.

**Fig 1:**
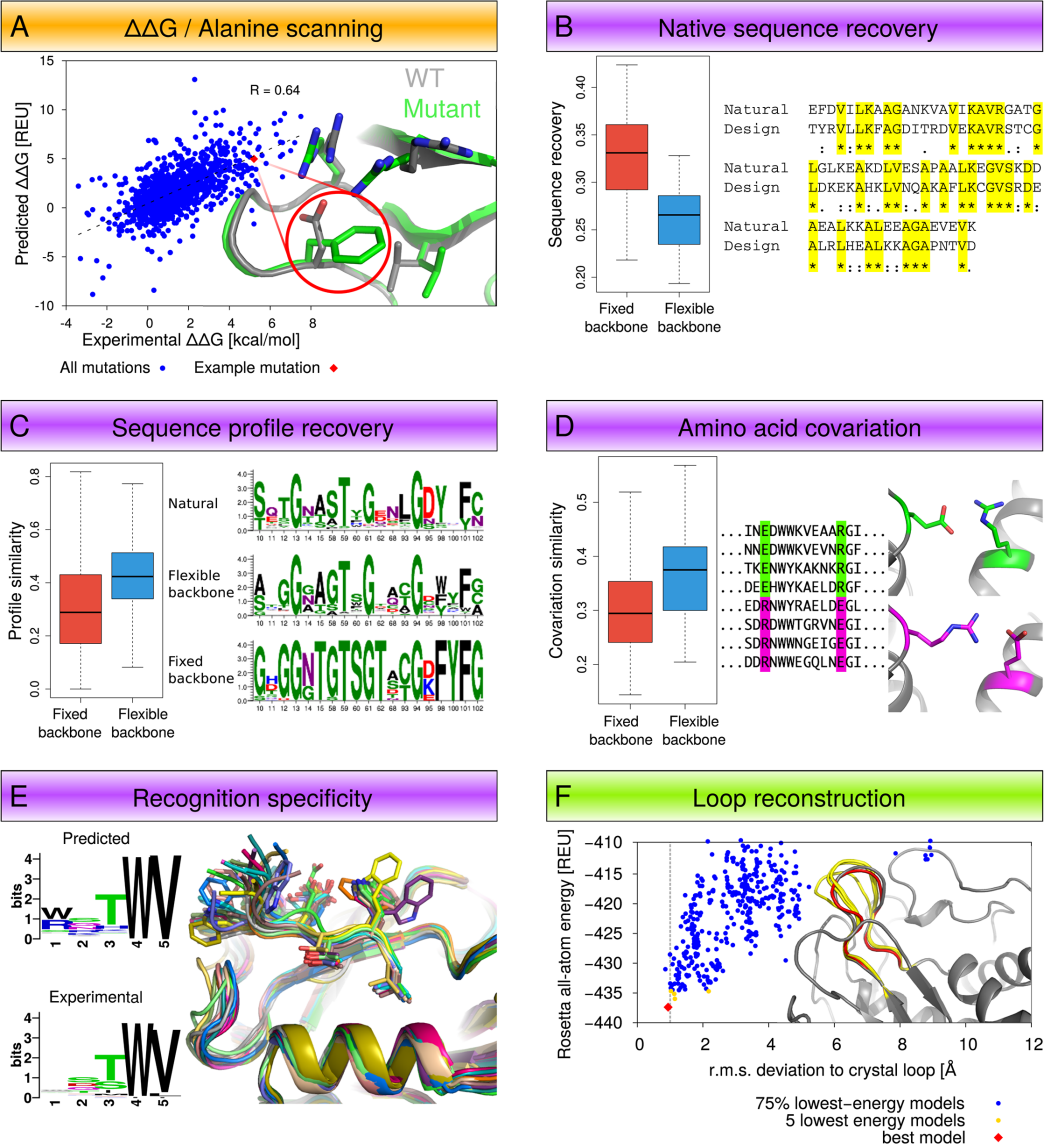
Types of benchmarks and protocols currently included in the web resource. Tests estimating energetic effects of mutation (orange, A), design tests (purple, B-E) and structure prediction tests (green, F).

We have used these guidelines to collect benchmark sets for commonly encountered problems in the following three areas (Fig 1): (1) estimation of energetic effects of mutations (protein stability (ΔΔG) and computational alanine scanning); (2) protein design predictions (native sequence recovery, evolutionary profile recovery, sequence covariation recovery, and prediction of recognition specificity); and (3) protein structure prediction (loop modeling). We also present corresponding state-of-the-art Rosetta protocols, parameters and command lines applicable to each problem. Each benchmark capture can be downloaded from the web resource either as a self-contained zip file/bundle or as a version-controlled repository. Each bundle contains the input data and documentation describing the given modeling problem, explains how the accompanying methods solve that problem, lists the metrics we use to measure success, includes the Rosetta protocol, and provides analysis scripts to generate these metrics from output data. In the sequel, we describe the technical details of the website we have created for open access and dissemination of benchmarking results.

(A) ΔΔG / Alanine scanning—predicting the energetic effect of point mutations on folding or binding. REU: Rosetta energy units. The dashed line represents the best linear fit model (y = 0.93x + 0.43). (B) Native sequence recovery—measuring the similarity between designed and native sequences for a given structure. Boxplots compare fixed to flexible backbone design performance, and designed residues that are identical to the native sequence are highlighted in yellow. (C) Sequence profile recovery—measuring the similarity between designed and natural sequence profiles of protein families. Boxplots compare fixed to flexible backbone design performance in recovering the natural sequence profile. (D) Amino acid covariation—predicting pairs of naturally covarying residues in protein families. Boxplots compare fixed to flexible backbone design performance, and covarying pairs in the multiple sequence alignment are highlighted in green and magenta. (E) Recognition specificity—predicting the tolerated sequence space in a protein-protein interface. The sequence logos [14] visualize the similarities and differences between the predicted and experimentally determined sequence profiles. (F) Loop reconstruction—predicting the backbone conformation of loops in protein structures. Here the scatterplot shows a minimum in the Rosetta energy landscape for the given loop, with the five lowest energy models shown in yellow and the one closest to the experimentally determined (native) structure highlighted in red.

## Benchmarks (Methods)

The web resource currently contains benchmark captures, Rosetta protocols, and performance information for five different benchmarks, which we have grouped below in three different areas (Fig 1): (1) tests estimating energetic effects of mutations, (2) design tests, and (3) structure prediction tests. Each subsection describes, for each benchmark, its purpose (the modeling problem addressed), the benchmark dataset, a Rosetta protocol addressing the modeling problem, metrics of success, key results, and notes on limitations and caveats.

### 1. Tests estimating energetic effects of mutation

#### Protein stability (ΔΔG)

Purpose of this test: The purpose of this benchmark is to predict the change in stability (ΔΔG) of a monomeric protein caused by single point mutations (Fig 1A). The predicted stability change is given as the difference in predicted energy between the modeled wild-type and mutant structures. The benchmark compares the predicted energy differences against experimentally measured ΔΔG values in kcal/mol.

Benchmark dataset: In previous studies, protein stability prediction methods have been benchmarked against multiple curated datasets: a set of 1030 mutants collected by Guerois et al. [15]; a set of 2156 mutants collected by Potapov et al. [16]; a set of 1210 mutants collected by Kellogg et al. [17]; and a set of 582 mutants collected by Benedix et al. [18]. The records in these datasets mainly originate from the ProTherm database [7]—a large, manually curated collection of thermostability data from the literature—and are mostly single point mutations. Our benchmark capture collects the Guerois, Potapov, and Kellogg datasets together and adds a fourth dataset of 2971 point mutants from ProTherm. This last dataset is lightly curated; it contains most of the single point mutations available in ProTherm excepting records where: (i) there is no corresponding structure determined via X-ray crystallography with a resolution of at least 2.5Å; (ii) there are multiple experimental ΔΔG values for an individual mutation that differ by more than 2.5 kcal/mol in the experimental values; or (iii) the mutated protein is a transmembrane protein.

ProTherm contains details of the publications from which the thermodynamic data originated. These explicit references were omitted in the previously published datasets mentioned above but we have determined the source of the mutations for each record in the benchmark capture and reformatted the datasets into a standardized format. This refactoring has allowed us to determine the overlap between the datasets in terms of mutations and experimental assays to a large degree. These refactored datasets are included in the benchmark capture.

Rosetta protocol: The benchmark capture currently includes scripts that can be used to run the best-performing protocol described by Kellogg et al. as protocol 16 (see row 16 in Table 1 in reference [17]). This protocol combines a soft-repulsive potential for conformational sampling of side-chains with a standard hard-repulsive potential for minimization to achieve higher prediction accuracy, following the observation that predictive methods are more accurate when the resolution of the force field is matched to the granularity of the sampling method. There are two steps in the protocol. First, the input structure is minimized. Next, fifty pairs of wild-type and mutant structural models are generated using the sampling strategy described above. The ΔΔG value is calculated as the difference between the three best-scoring wild-type structural models and the three best-scoring mutant structural models as measured in Rosetta energy units (REU).

Performance metrics: Three metrics are used for measuring the accuracy of the computational methods, each with a separate focus.

Pearson's correlation coefficient measures the linear correlation between experimentally determined ΔΔG values and their corresponding computationally predicted values. The coefficient is invariant to the scale of the predicted values.

The mean absolute error (MAE) is defined as the mean of the absolute differences between experimental and predicted ΔΔG values. MAE is sensitive to the scale of the predicted values and is an important metric for protein design; high error reduces confidence in the predicted stability of individual cases.

Finally, the *stability classification accuracy* or *fraction correct* metric measures whether a mutation is correctly predicted to be (de)stabilizing or neutral, for a given definition of what constitutes a neutral mutation. Depending on this definition, it is possible to get a relatively high value for this metric with a set of random predicted values. Therefore this metric, while a useful metric for reporting whether a method can correctly classify the stability of a mutant, should be considered alongside the correlation and MAE.

Key results: It has been previously reported that the latest Rosetta score function (Talaris) improves the performance of the Rosetta ΔΔG protocol on the Kellogg dataset compared to the older score function, termed Score12 [19]. We have tested the protocol on the three other curated datasets and found that Talaris improves the correlation with comparable MAE values for these datasets as well, compared to Score12. However, the performance measured by the same metric differs significantly between the different datasets, suggesting that the datasets represent different levels of prediction difficulty. These data are presented on the website.

Notes: (i) We have made some modifications to the datasets from the original publications, such as updating deprecated PDB identifiers and correcting PDB IDs, PDB residue IDs, and ΔΔG values based on cross-referencing to the respective publications. We now attribute each record of a dataset with publications from which the ΔΔG values originate. This information was not present in some of the published datasets. (ii) Neutral experimental ΔΔG values are defined as values within +/-1 kcal/mol, as used by Kellogg et al. We define neutral predicted ΔΔG values as values within +/-1 score unit which differs from their definition (see [17] supporting information; neutral predicted is defined to be in the range [-3, 1.1]).

#### Alanine scanning

Purpose of this test: A frequent application of modeling methods is the prediction of energetically important interactions (“hotspots”) in protein-protein interfaces. By systematically mutating protein interface residues to alanine (“alanine scanning”) and measuring the effect on binding, Wells and coworkers [20] showed that not all residues with interface contacts, but only a smaller subset of ‘hotspot’ residues contribute significantly to the binding free energy of human growth hormone to its receptor. Subsequent studies suggested that such hotspots may be a general characteristic of many protein-protein interfaces [21–23]. This benchmark tests the ability of computational alanine scanning protocols to recapitulate the results of measurements of changes in binding affinity (ΔΔG values) produced by experimental alanine scanning. A computational protocol performing well on this test set can then be used for additional applications, for instance, as a design tool to disrupt protein-protein interactions by mutations or through targeting small molecules to hotspots, or to analyze the effect of disease mutations.

Benchmark dataset: The protocol has been benchmarked on a previously published set of the energetic effects of 233 mutations to alanine in 19 different protein-protein interfaces with known crystal structures [24].

Rosetta protocol: We have re-implemented a previously published alanine scanning protocol [24,25] in the current version of Rosetta to determine the current performance of this method. Unlike the generalized ΔΔG protocol described above, which performs side chain optimization and side chain and backbone minimization over the entire protein structure, the alanine scanning protocol does not model perturbation of the backbone or side chains other than the side chain of the residue replaced with alanine.

The ΔΔG of binding upon mutation to alanine is calculated using the following equation, in which Rosetta total energy is used to estimate the ΔG of folding of each of the six terms:

$$\Delta \Delta G_{bind}=(\Delta G_{complex}^{MUT}-\Delta G_{partnerA}^{MUT}-\Delta G_{partnerB}^{MUT})-(\Delta G_{complex}^{WT}-\Delta G_{partnerA}^{WT}-\Delta G_{partnerB}^{WT})$$

Alanine scanning uses a version of Rosetta’s Talaris energy function with modified weights intended for scoring mutations to alanine within interfaces, where the score term representing repulsive electrostatic interactions is down-weighted.

The previously published protocol [24,25] is available via the Robetta webserver at http://robetta.bakerlab.org/alascansubmit.jsp, which has provided more than 20,000 predictions to date. The implementation described here will allow users to run predictions off-line and on large datasets, and implement and test modifications to the protocol.

Performance metrics: Performance can be measured using the same metrics as in the generalized case of the ΔΔG protocol described above, including the Pearson’s correlation of predicted ΔΔG values to experimental ΔΔG values, mean absolute error (MAE), and fraction correct (see previous section for descriptions of these metrics).

Key results: Alanine scanning performance has not shown improvement when used with modern Rosetta score functions and aggressive side chain/backbone minimization methods; performance of the protocol described here is comparable to that shown in earlier publications [24,25] and available on the Robetta server.

Notes/Limitations: (i) As the alanine scanning protocol does not perturb the protein backbone or side chains (other than the mutant residue), this protocol is not suitable for use on mutations outside of the interface. A mutation outside of the interface will not change the predicted interaction energy without the use of a more intensive sampling protocol. (ii) As the backbone structure of the wild-type crystal structure is assumed to be a close approximation of the backbone structure of the mutant, this protocol is not useful in situations where this assumption does not hold. This includes testing of many simultaneous mutations that may result in larger structural rearrangements.

### 2. Design tests

Protein design methods are difficult to test rigorously because an ideal benchmark set would contain both successful and unsuccessful designs, however, the number of cases where both have been characterized functionally and structurally is small and not yet diverse enough. Until the amount of available data of this nature greatly increases, other datasets, in particular the diversity of sequences present in naturally evolved protein families or selected in large-scale experimental screens, can provide informative benchmarks that have been used in the past to assess and compare design methods [26,27]. In the following sections, we first focus on design tests using evolutionary information, and then describe a benchmark testing prediction of protein recognition specificity using data from comprehensive phage display experiments. In each case, we compare designed and evolutionary or experimentally selected sequences using metrics comparing not individual sequences (as the number of possible sequences is large and hence the chance of an exact match at all sequence positions extremely small), but instead predicted and observed amino acid distributions.

### 2.1 Using evolutionary information

#### Purpose of this test

Evolutionary pressures on protein structure and function have shaped the amino acid sequences of today's naturally occurring proteins [28]. Consequently, the sequences of natural proteins are nearly optimal for their structures [29]. Natural protein sequences therefore provide valuable information for evaluating the accuracy of computational protein design in predicting sequences consistent with a given protein structure and function. We expect that an ideal computational protein design method should be able to recapitulate properties of naturally occurring proteins, including amino acid sequence preferences (“sequence profiles”) and patterns of amino acid covariation. In particular the latter tests whether computational protein design methods are capable of recapitulating the precise details of specific residue-residue interactions in proteins.

#### Benchmark dataset

To evaluate to what extent protein design methods can recapitulate properties of naturally evolved proteins, we first characterized amino acid sequence profiles and amino acid covariation in 40 diverse protein domain families. Protein domains for this benchmark were selected from Pfam [30] based on the following criteria: (i) there is at least one crystal structure of the domain available from the PDB; (ii) there were at least 500 sequences of the domain family available from Pfam; and (iii) the domain had 150 or fewer amino acids. We selected 40 structurally diverse domains that satisfied these criteria. Sequence profiles were calculated by determining the amino acid distribution at each position and amino acid covariation was calculated for all pairs of amino acids using a mutual information based metric [31].

#### Rosetta protocol

We designed 500 sequences for each domain using a variety of protein design methods that used the same energy function but differed in how they modeled protein backbone flexibility. As a baseline, we performed fixed backbone protein design, which does not allow the backbone to be moved. Flexible backbone design simulations were performed multiple times using different temperatures and different types of backbone moves to assess how the magnitude and mechanism of backbone variation affects the recapitulation of natural sequence properties. The different types of backbone moves included Backrub, Kinematic Closure (KIC), small phi/psi moves and all atom minimization (Relax). We also tested fixed backbone design using a soft-repulsive energy function. Additional details on the different methods are described in [26] and Rosetta command lines are provided on the web resource, along with a performance comparison.

#### Notes/Limitations

This benchmark makes the assumption that naturally occurring proteins are optimized for stability given their particular three-dimensional structures used as input. However, there certainly exist cases where proteins trade stability for function, such as hydrophobic patches that act as protein-protein binding interfaces or charged residues in the protein core used for catalyzing chemical reactions. We therefore expect to observe some differences between naturally occurring sequences and sequences predicted by an accurate protein design method (even if it were perfect). The benchmark assumes that methods that predict more “native-like” sequences overall are more accurate and thus more useful for experimental design applications [29]. In these applications, functional constraints, such as binding and catalysis, are usually explicitly represented by including functional binding partners or specifying certain key catalytic groups and their conformations.

### Native sequence recovery

#### Performance metrics

Native sequence recovery [29] measures the ability of computational protein design to predict the amino acid sequence of a protein given its backbone conformation (Fig 1B). This is simply calculated as the percent identity between the native sequence and a designed sequence.

#### Key results

We found that adding a small degree of backbone flexibility prior to design increased sequence recovery on average, however, further increasing the amount of backbone flexibility led to worse sequence recovery scores. A possible explanation for this decrease in recovery is that allowing more backbone flexibility resulted in sequences with a greater diversity in their amino acid sequences and consequently greater divergence from the native sequence. To confirm this, we calculated sequence entropy for the designed sequences and found that structural variation is positively correlated with sequence diversity. These results highlight a caveat with using native sequence recovery as a test of protein design accuracy, which is that protein sequences can be very different from each other but still be consistent with the same protein fold [32], and it is this sequence divergence that can be utilized to evolve existing proteins for new functions.

### Sequence profile recovery of protein families

#### Performance metrics

Sequence profiles represent the distribution of amino acids at each position in a multiple sequence alignment of a protein family (Fig 1C). To compare natural and designed sequence profiles, we computed the divergence between the amino acid distributions at corresponding positions in the natural and designed sequences, as described in [32]. Briefly, profile similarity is the product of two scores: (i) the estimated probability that two amino acid distributions represent the same source distribution; and (ii) the *a priori* probability of the source distribution. It is defined as:

$$Profile\;Similarity(p,q)=\frac{1}{2}(1-D^{JS}[p\|q])(1+D^{JS}[r\|P_{0}])$$

where p and q are amino acid probability distributions at corresponding positions in natural and designed sequences, r is the average of p and q, P_0_ is the background distribution, and D^JS^ is the Jensen-Shannon divergence. Using this metric, positions in designed sequences receive high profile similarity scores if both: (i) their amino acid distribution is similar to the amino acid distribution at the corresponding position in the natural alignment; and (ii) their amino acid distribution is different than the background amino acid distribution.

#### Key results

**Fig 2:**
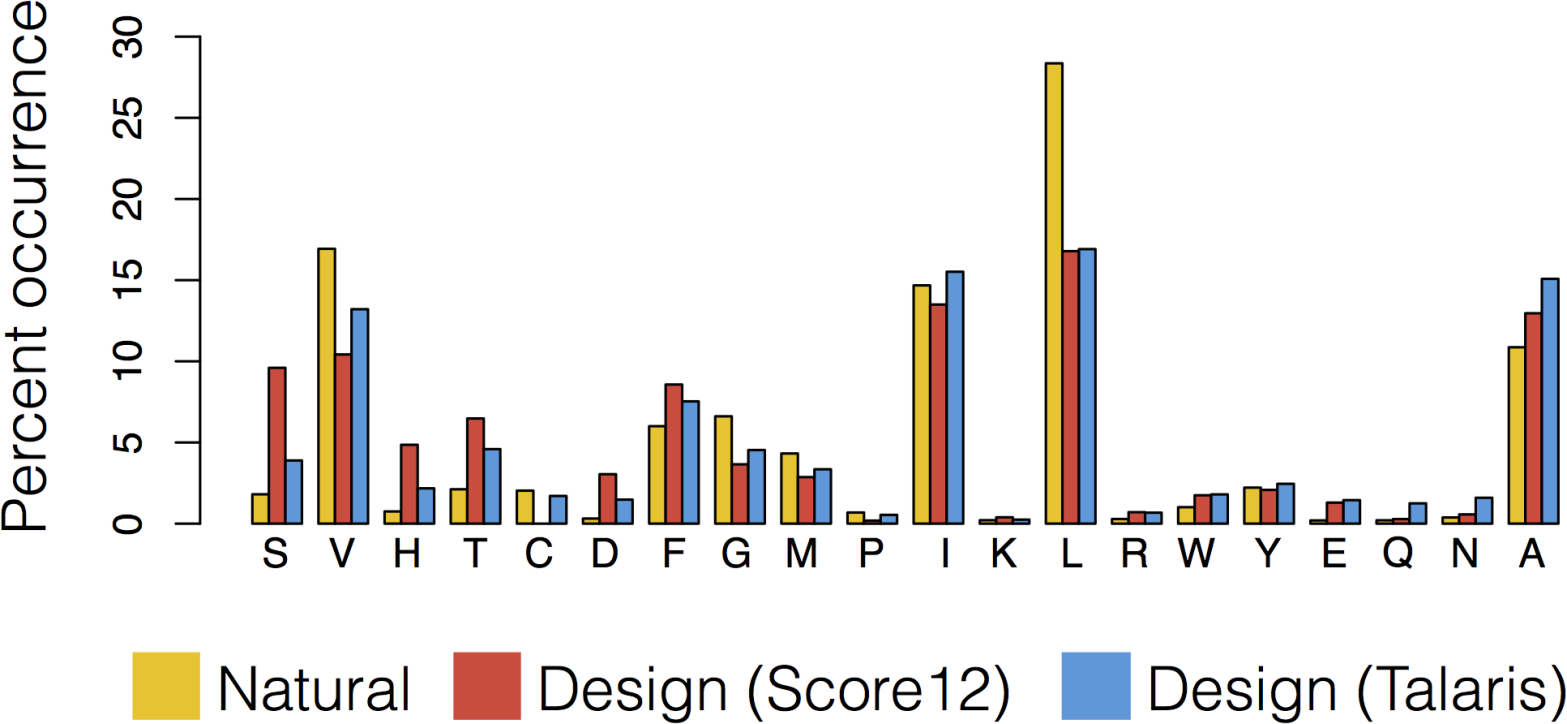
Comparison of occurrences of different amino acid residue types observed at buried positions between natural sequences and sequences designed with two different Rosetta energy functions. Barplot showing the percent occurrence of each type of amino acid found at buried positions in natural and designed sequences across 40 diverse protein families. Buried positions are defined as positions with greater than 14 neighboring positions, where neighboring positions have C-β atoms within 8Å of the C-β atom of the residue of interest. The X-axis is sorted by the magnitude of improvement of the Talaris energy function relative to the previous Score12 energy function with respect to the similarity to the natural percent occurrences.

We observed that backbone flexibility improved our ability to recapitulate sequence profiles of naturally occurring protein families relative to fixed backbone design, and that there exists an optimal magnitude of backbone flexibility (using Rosetta kT = 0.9 in “backrub” simulations, [26]) given that low or high temperature simulations performed worse than medium temperature simulations. This analysis also revealed an important pathology in the designed sequences, which showed an unrealistically high percentage of designed buried polar residues when compared to the natural sequences. To overcome this problem, we repeated the benchmark using a newer Rosetta energy function, Talaris [19] that has stricter definitions for hydrogen-bonding geometries. We found that this decreased the percentage of buried polar residues, including serine, threonine and histidine, although it remained higher than in the natural sequences (Fig 2 and S1 Fig). These results can be quite sensitive to the reference energies in the applied energy function (which allow energetic evaluation of mutations). Existing automated tools [33] that reweight reference energy terms can be used to develop alternative energy functions. Future improvements to sampling and scoring will be required to further reduce the percentage of buried polar groups to levels found in naturally occurring proteins.

### Amino acid covariation

#### Performance metrics

To evaluate how well a given protein design method could recapitulate natural amino acid covariation, we designed 500 sequences for each protein domain in the benchmark and calculated the covariation between all pairs of positions in the designed sequences (Fig 1D). Covariation is calculated based on a mutual-information based metric described in [31]. The mutual information (MI) between each pair of columns in a multiple sequence alignment, i and j, was calculated as the difference between individual entropies (H_i_, H_j_) and the joint entropy (H_i,j_):

$$MI_{i,j}=H_{i}+H_{j}-H_{i,j}$$

The background mutual information due to random noise and shared ancestry is then subtracted to obtain the product corrected mutual information (MIp) [34]:

$$MIp_{i,j}=MI_{i,j}-\frac{{\bar{MI}}_{i}\times {\bar{MI}}_{j}}{\bar{MI}}$$

where ${\bar{MI}}_{j}$ is the mean MI of position i with all other positions and $\bar{MI}$ is the overall mean value. Next this value is converted to two Z-scores, one for each column, which are then multiplied together:

$$Z_{i\times y}=\frac{MIp_{i,j}-\bar{MIp_{i}}}{\sigma (MIp_{i})}\times \frac{MIp_{i,j}-\bar{MIp_{j}}}{\sigma (MIp_{i})}$$

The final covariation score, called Zpx, is calculated as the square root of the absolute value of *Z*_*i*×*y*_. (If *Z*_*i*×*y*_ is negative, then Zpx is multiplied by-1.) This normalization was previously shown to reduce sensitivity to potential misaligned regions in multiple sequence alignments, which otherwise result in artificially high MI scores [31]. Similarity between natural and designed covariation was calculated as the percent overlap between the highly covarying pairs in the natural sequences and in the designed sequences. We considered pairs with covariation scores greater than two standard deviations from the mean to be highly covarying [26].

#### Key results

We used this metric for quantifying the similarity of natural and designed covariation in order to compare different flexible backbone protein design methods that varied in either the magnitude or mechanism of backbone flexibility. As with sequence profile recovery, we observed that backbone flexibility improved our ability to recapitulate naturally occurring amino acid covariation relative to fixed backbone design, and that there exists an optimal magnitude of backbone flexibility (in the range of kT = 0.6 to kT = 0.9 in Rosetta simulations). We also found that flexible backbone design methods which incorporate backbone flexibility via iteratively applying local backbone moves (*e*.*g*. Backrub [35] or Kinematic Closure [36]) performed better than Rosetta methods that globally alter the backbone of the entire protein (*e*.*g*. Relax or AbInitioRelax) [26].

### 2.2 Using large-scale experimental data

#### Recognition specificity

Purpose of this test: “Sequence tolerance” refers to the concept that a certain profile of allowed residues can accommodate the evolved structure and function of a protein (Fig 1E). The computational sequence tolerance protocol attempts to predict the allowed sequence profile in protein-protein interfaces. The predictions are tested for their ability to recapitulate the sequence specificity preferences of protein recognition domains that have been determined by comprehensive phage display experiments. In contrast to the comparison to sequences of evolutionary families in the previous section, the experimentally determined profiles were selected primarily based on the same criterion (most stable binding) as in the design simulations.

Benchmark dataset: The experimental data used for comparison in this benchmark set come from phage display specificity profiles for naturally occurring PDZ domains [37], as well as phage display profiles for peptide interactions with synthetic variants of the Erbin PDZ domain [37,38], comprising over 8000 peptide sequences tested against 169 natural and synthetic PDZ domains total.

Rosetta protocol: There are two main computational steps: (i) the Rosetta Backrub application [35] uses Monte Carlo sampling starting from a single input structure to create an ensemble of near-native conformations; (ii) the sequence tolerance application [39,40] then uses a genetic algorithm to sample and score a large number of sequences for each member of the ensemble. An input file defines the sequence positions to be designed, and interactions within and between different parts of the structure can be individually reweighted, depending on the desired objective.

Performance metrics: The analysis scripts use Boltzmann weighting to generate a predicted position weight matrix (PWM) for the specified sequence positions. This predicted PWM can be compared to known sequence profiles via these metrics described in the previous sequence tolerance publications [39,40]: (i) AAD, average absolute deviation, defined as $\frac{1}{N}\sum_{i=1}^{N}|E_{i}-P_{i}|$ and (ii) Frobenius distance, defined as $\sqrt{\sum_{i=1}^{N}{\left(E_{i}-P_{i}\right)}^{2}}$, where *E* is the vector of experimentally determined amino acid frequencies and *P* is the corresponding vector of predictions. (iii) AUC, or area under the receiver operator characteristic curve, measures the ability of the predictions to match the experimental values on a known scale, where 0.5 indicates random predictions and 1.0 is perfect. (iv) “Rank top” measures the predicted rank of the most frequent experimentally determined amino acid.

Key results: Recognition specificity performance with Talaris is comparable to the originally published performance of the protocol with Score12 [39,40].

Notes/Limitations: (i) Although the sequence tolerance protocol is capable of generating backbone flexibility, which improves performance, it still relies on known input starting structures. Mutations can be made to these starting structures in order to predict the recognition specificity of experimentally characterized mutated proteins, but the additional mutation step might reduce the overall performance of the protocol. (ii) The backrub phase of the protocol must be run at a reasonable temperature (see protocol capture) to generate an appropriately matching amount of backbone flexibility in the sequence tolerance step. (iii) Due to limitations in the sequence space sampled by the genetic algorithm, it is not recommended to try and sample more than about 4–6 design positions simultaneously. (iv) Sequence profiles produced by this method may accurately predict the most frequently observed amino acid at a design position without containing enough total variation at that same position. (v) The performance metrics described above ignore potential co-variation in predicted or experimentally selected sequences.

### 3. Structure prediction tests

#### Loop reconstruction

Purpose of this test: Being able to correctly model loop conformations (Fig 1F) is crucial because of their functional importance in many proteins, such as in forming the complementarity-determining regions in antibodies or in controlling substrate access and product release in enzyme active sites. However, since many loops in protein structures are flexible, loop modeling is computationally hard, because the many backbone degrees of freedom (depending on the length of the loop) result in a vast conformational search space. The purpose of this test is to reconstruct known native loop conformations, as observed in crystal structures, in non-redundant benchmark sets of different loop lengths.

Benchmark dataset: The Rosetta loop modeling benchmark [36,41] tests the ability of a protocol to reconstruct the backbone conformation of 12-residue loop segments in protein structures. The benchmark set consists of 45 non-redundant protein segments without regular secondary structure, curated from two previously described datasets [42–47]

In each case, the given segment is deleted from the protein structure and then reconstructed *de novo*, given a fixed backbone environment for the rest of the protein. All segment side chains and those within 10Å of the segment are modeled based on a side chain rotamer library [48] that does not include the native side chain conformations.

The long loops benchmark [41] analogously tests whether protocols are able to reconstruct loop segments of 14–17 residues. This benchmark set consists of 27 non-redundant long loops, extracted and manually curated from the dataset described in [49], by requiring at most five residues within 6Å of symmetry mates in the crystal lattice to minimize the potential impact of crystal contacts on loop conformations. *De novo* loop reconstruction and side chain optimization are performed as described above for the standard loop modeling benchmark.

Rosetta protocol: Several protocols have previously been developed to reconstruct or predict the backbone conformation of loops in protein structures. The CCD protocol in Rosetta [43] uses insertion of fragments from proteins of known structure to sample the loop backbone degrees of freedom, followed by torsion angle adjustments via cyclic coordinate descent (CCD) to close the resulting chain break [50]. The kinematic closure (KIC) protocol [36] samples all but six loop backbone degrees of freedom probabilistically from Ramachandran space. These remaining three pairs of φ/ψ torsion angles are then solved analytically through kinematic closure to close the chain break [51]. Next-generation KIC (NGK) [41] adds four additional sampling strategies to the standard KIC protocol: (i) the selection of pairs of φ/ψ torsions from neighbor-dependent Ramachandran distributions; (ii) sampling of ω degrees of freedom; as well as annealing methods that gradually ramp the weights of (iii) the repulsive terms; and (iv) the Ramachandran terms of the Rosetta energy function to overcome energy barriers. All three loop modeling protocols use Monte-Carlo simulated annealing for rotamer-based side-chain optimization (“repacking”) of the loop residues and those within 10 Å of the loop, followed by gradient-based minimization.

Performance metrics: With each loop modeling protocol, hundreds of models are generated per benchmark case. Each model is then superposed onto the native structure (excluding the reconstructed loop), followed by calculating the loop backbone heavy-atom root mean square deviation (RMSD) of the model to the native loop conformation. The overall benchmark performance of each protocol is then evaluated using two different metrics across the entire benchmark set: (i) the median loop backbone RMSD of the lowest-energy model to the native structure (or median lowest loop backbone RMSD of the 5 lowest-energy models, which is less susceptible to stochastic fluctuations [33]); and (ii) the median percentage of models generated that have a loop backbone RMSD below 1Å (sub-angstrom predictions).

Key results: With the Rosetta Score12 energy function (the standard before the switch to the Talaris2013 energy function in revision 55274), only the KIC [36] and NGK [41] protocols successfully sampled sub-angstrom loop conformations in many cases, achieving a median RMSD across the entire 12-residue loop benchmark set of <1Å. NGK significantly outperformed standard KIC in the sampling of sub-Å loop conformations, with NGK reaching a median of 16.3% sub-Å models compared to 4.3% for standard KIC [41]. Since the advent of the Talaris2013 score function [19,33], the CCD protocol now also achieves a median RMSD < 1Å on the 12-residue loop dataset. The median percentage of models with sub-Å RMSD is still significantly higher for NGK (13.4%) than for standard KIC (6.4%) and CCD (1.8%). For the more difficult sampling problem in the long loops benchmark, the sub-Å sampling performance of NGK improved from Score12 (0.53%) to Talaris2013 (1.0%).

Notes/Limitations: (i) Flexible loops are often better described by a conformational ensemble rather than a single conformation, and some simulations indeed reveal several clusters of different low-energy conformations. (ii) Crystal contacts can influence loop conformations, and the absence of those contacts during modeling can result in predictions differing from the crystallographic conformation. (iii) There are similar considerations for the presence of water molecules, ions or other small molecules, which might influence loop conformations. (iv) For the KIC and NGK protocols, the start and end points of loops are assumed fixed during the simulations; this simplifies the sampling problem in the context of “native” loop endpoints (*i*.*e*. taken from a crystal structure), but complicates the situation when the conformation of loop endpoints may not be known exactly, *e*.*g*. when building loops in homology models. In these cases, protocols that sample the positions of the endpoints or apply KIC moves over several overlapping regions may be more suitable. (v) Modeling long loops is difficult for current protocols, due to the large conformational search space, which is apparent from the considerably lower fraction of sub-angstrom models in the 14–17 residues loop benchmark. (vi) The KIC and NGK protocols do not preserve protein secondary structure, due to probabilistic sampling of φ/ψ torsions from Ramachandran space. Additional sampling constraints could be included to preserve secondary structure.

## Website Description

The benchmark captures are collected and presented online at https://kortemmelab.ucsf.edu/benchmarks. The purposes of the website are to: (i) describe specific and well-defined problems in computational modeling; (ii) describe and provide benchmarks which can be used to measure the success of methods designed to address these problems; (iii) publish the performance of methods using parameters provided by experienced users; and (iv) act as a unified portal for downloading the benchmark captures.

**Fig 3:**
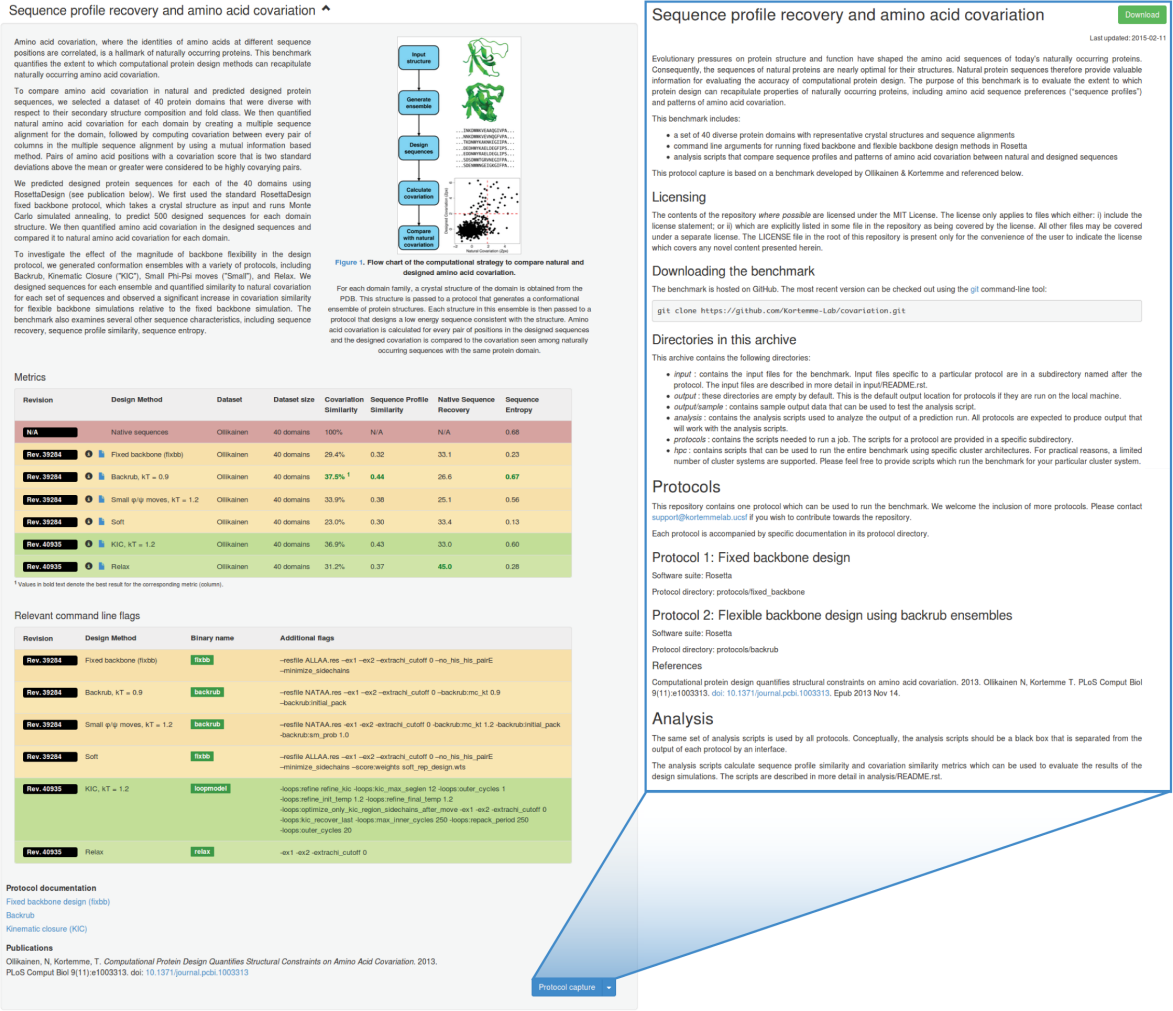
Benchmark and protocols capture website. Left: The website presents an overview of each benchmark and publishes the performance of different methods using a set of standardized metrics. Parameters important to the protocol performance are also provided. Right: Each benchmark capture is stored in a documented version-controlled archive. The most recent version can be downloaded directly from the website.

On the main benchmark page (Fig 3A), we describe each benchmark—its purpose, application, and the currently considered datasets—and publish results of benchmark runs so that users can quickly gauge the performance of different methods. Relevant command lines are provided to promote best practice for each method when using the Rosetta software suite.

Each capture has been compiled as a version-controlled, publicly-accessible, open-source archive (currently hosted on GitHub), containing both the associated benchmark datasets and scripts to analyze benchmark output in a specific format. Execution scripts to run the benchmark using at least one computational method are provided. Both the analysis and execution scripts are documented in detail within the capture, and this documentation can be viewed online on GitHub. For convenience, these captures are available for download directly from the web resource (Fig 3B).

As our intention is to provide a dynamic resource, it may be appropriate to refine or expand certain datasets as new data become available in the future. Version control allows us to update the contents while allowing users to track changes in the datasets or analysis metrics. Major changes to repositories will be tagged and referred to in the website text. Following the philosophy of the computer language benchmark projects [52,53], the parameters used for each method should reflect the best practice. For this reason, they should be ideally contributed by a developer or experienced user, and we encourage users to submit their methods, parameters, and results for inclusion on the website.

We have aimed to provide rich, user-friendly datasets. For example, the protein stability datasets are provided in both JSON and CSV formats. The former is readily integrated with multiple programming languages and web frameworks whereas the latter is human-readable and easily imported into spreadsheet applications. In both of these datasets, each record is now associated with experimental values taken from the literature—which we were able to determine using the rich source of data provided by the ProTherm database [7]—so that outliers in the predicted set can be investigated using the original experimental data.

## Discussion

We have presented our implementation of a benchmarking and protocol capture web resource which currently describes five diverse benchmarks and their expected performance when tested using a known best-practice methods from the Rosetta software suite. The web site functions as an openly accessible, online, and version-controlled collection of a variety of benchmarks and macromolecular modeling and design protocols, providing a summary of the evolution of the protocols and indicating their expected performance on the associated benchmarks.

The web resource was motivated by previous work [52,53] which has fostered, and continues to foster, competition and innovation in computer language development through the open communication of standardized benchmarks which allow for direct and fair comparison between competing computer languages. In those projects, knowledge of the performance of each language for the particular problem and the open communication of the most efficient code is important for both software developers when choosing which language to use for a particular project and for the language developers so that they can identify parts of the language for optimization. By providing both curated diverse datasets for benchmarking and analysis scripts to generate a set of appropriate metrics, we hope that we can help developers to evaluate new methods in informative ways, which is critically needed for continued progress in many areas of structure-based modeling and design.

## Supporting Information

S1 Fig Comparison of occurrences of different amino acid residues (by polarity) observed at buried and exposed positions. Each barplot shows the percent occurrence of each polarity category of amino acid found in natural and designed sequences across 40 diverse protein families. Yellow bars show the percent occurrence in natural sequences, red the percent occurrence in sequences designed using Rosetta’s Score12 energy function, and blue the percent occurrence when designing with Rosetta’s Talaris energy function. Neighboring positions are defined as any position with a C-β atom within 8Å of the position being investigated. For the purposes of this figure, nonpolar amino acids are defined as: CGAVLIMFWP, polar: STYNQ, charged: HRKDE. (A) Exposed positions are defined as positions with between 0 and 8 neighboring positions. (B) Buried positions are defined as positions with greater than 14 neighboring positions. Using the Talaris energy function reduces the percentage of charged residues placed in buried positions by 43% (from 10.5% to 6.2%), bringing the predictions closer to the native sequence properties.
(EPS)
